# Piceatannol Upregulates SIRT1 Expression in Skeletal Muscle Cells and in Human Whole Blood: In Vitro Assay and a Randomized, Double-Blind, Placebo-Controlled, Parallel-Group Comparison Trial

**DOI:** 10.3390/life14050589

**Published:** 2024-05-05

**Authors:** Kenta Tanaka, Shinpei Kawakami, Sadao Mori, Takumi Yamaguchi, Eriko Saito, Yuko Setoguchi, Yuko Matsui, Eisaku Nishimura, Shukuko Ebihara, Toshihiro Kawama

**Affiliations:** 1 R&D Institute, Morinaga & Co., Ltd., 2-1-1 Shimosueyoshi, Tsurumi-ku, Yokohama 230-8504, Japan; k-tanaka-bb@morinaga.co.jp (K.T.); s-kawakami-jf@morinaga.co.jp (S.K.); s-mori-ab@morinaga.co.jp (S.M.); t-yamaguchi-bc@morinaga.co.jp (T.Y.); e-saito-jd@morinaga.co.jp (E.S.); y-setoguchi-ia@morinaga.co.jp (Y.S.); y-matsui-jd@morinaga.co.jp (Y.M.); e-nishimura-ie@morinaga.co.jp (E.N.); 2 Chiyoda Paramedical Care Clinic, 3-3-10 Hongokucyo, Nihonbashi, Cyuo-ku, Tokyo 103-0021, Japan

**Keywords:** piceatannol, passion fruit seed, fat metabolism, SIRT1, mitochondria, resveratrol, skeletal muscle

## Abstract

Piceatannol (PIC), a polyphenol abundant in passion fruit seeds, is reported to promote fat metabolism. This study investigated whether PIC affects sirtuin 1 (SIRT1) expression and metabolic factors in C2C12 skeletal muscle cells. C2C12 myotubes were stimulated with PIC, and alterations in gene expression, protein levels, mitochondrial DNA content, and fatty acid levels were assessed using real-time PCR, Western blotting, and Nile red staining. Furthermore, we examined changes in SIRT1 expression following the consumption of a test food containing 100 mg PIC for 2 weeks among adults with varying age and body mass index ranges. Both PIC and passion fruit seed extract induced SIRT1 expression in C2C12 myotubes to a greater extent than resveratrol. PIC also increased the expression of genes associated with mitochondrial biogenesis and fatty acid utilization, increased mitochondrial DNA content, and suppressed oleic acid-induced fat accumulation. Moreover, participants who consumed PIC exhibited significantly higher SIRT1 mRNA expression in whole blood compared to those in the placebo group. These findings suggest that PIC induces SIRT1 expression both in vitro and in the human body, which may promote mitochondrial biosynthesis and fat metabolism.

## 1. Introduction

Healthy life expectancy refers to the average number of years that a person can expect to live in “full health” by considering the years lived in less than “full health” due to disease or injury [1]. According to the World Health Organization, the global average life expectancy increased from 66.8 to 73.4 years, with the healthy life expectancy being extended from 58.3 to 63.7 years in the past two decades (2000–2019) [2]. Although both life expectancy and healthy life expectancy are increasing, the gap between the two has widened from 8.5 to 9.7 years, indicating that although people live longer, they are not necessarily spending these additional years in good health or being physically active. Hence, it is crucial to address this issue to enable people to enjoy longevity while leading healthy and fulfilling lives, and to tackle societal challenges such as rising healthcare costs and increased demand for care in an aging society. Sirtuin genes, particularly sirtuin 1 (SIRT1), are well-conserved nicotinamide adenine dinucleotide (NAD+)-dependent deacetylases that deacetylate acetylated lysine residues in numerous histone and nonhistone proteins, and SIRT1 is crucial for the aging process and metabolism [3,4,5,6]. SIRT1 was first identified as a factor that extends the lifespan of various organisms, including yeast [7], fruit flies [8], *Caenorhabditis elegans* [9], and mice [10,11]. SIRT1 regulates metabolism in different tissues [12], including promoting gluconeogenesis in the liver [13] and reducing fat accumulation and increasing free fatty acid levels in the white adipose tissue (WAT) [14]. Moreover, SIRT1 enhances insulin secretion in the pancreas [15,16] and insulin sensitivity [17,18,19]. Conversely, decreased SIRT1 expression has been reported in individuals with metabolic syndrome, insulin resistance, or obesity [20,21]. SIRT1 activation leads to the prevention of various chronic diseases associated with aging [12,22,23]. Recently, research on nicotinamide mononucleotide (NMN) has attracted attention as a means of enhancing NAD+, the cofactor of SIRT1 [24]. SIRT1 is increasingly gaining attention as a factor with the potential to improve a healthy lifespan by helping to prevent a variety of age-related disorders.

SIRT1 is an important regulator of mitochondrial biogenesis, fatty acid oxidation, and energy expenditure in skeletal muscles [25,26]. Hence, SIRT1 is fundamental for maintaining energy homeostasis, enhancing muscle fiber strength, and facilitating recovery from injuries [27]. Additionally, mitochondrial dysfunction in skeletal muscles is central in the development of various metabolic diseases, such as obesity and associated metabolic disorders [26]. For instance, SIRT1 overexpression attenuated high glucose-induced insulin resistance by reducing mitochondrial dysfunction in skeletal muscle cells [28]. Moreover, the administration of the SIRT1 activator SRT1720 enhanced the endurance running performance and protected mice against insulin resistance and diet-induced obesity by enhancing oxidative metabolism in their skeletal muscle and other tissues [29]. These studies suggested that SIRT1 activation in the skeletal muscles may contribute to preventing metabolic disorders and obesity.

Piceatannol (PIC), a plant-derived polyphenolic compound with a stilbene structure that is abundant in passion fruit seeds [30,31], has antioxidant [32] and anti-inflammatory properties [33], inhibits fat adipogenesis [34], and reduces cognitive impairment [35]. In clinical trials, the intake of PIC from passion fruit seeds helped maintain skin hydration and elasticity [36,37] and increased fat burning at rest and during moderate exercise [38,39]. PIC increased the expression of heme oxygenase-1 (HO-1) and superoxide dismutase 1 (*SOD-1*) and reduced the accumulation of H_2_O_2_-induced reactive oxygen species (ROS) in skeletal muscle cells [32]. Furthermore, PIC increased glucose uptake into skeletal muscle cells by promoting the translocation of GLUT4 to the cell membrane [40]. Collectively, these findings suggest that PIC plays a considerable role in reducing oxidative stress and influencing glucose metabolism in skeletal muscles. Resveratrol (RES), a compound related to PIC, is present in plants such as peanuts, cocoa, and berries [41,42,43]. This polyphenol, notable for its antiaging benefits [44], has been extensively studied for its various physiological effects. Human clinical studies targeting patients with coronary heart disease and type 2 diabetes mellitus have revealed that RES intake increased SIRT1 levels in the blood [45]. Although RES has been extensively studied, few reports exist on PIC [46], especially regarding its effect on SIRT1 expression in skeletal muscles and its potential to induce SIRT1 expression in humans when consumed. 

Here, we examined the effects of PIC on SIRT1 expression in the skeletal muscle cell line C2C12 and in adults across a broad range of age and body mass index (BMI) who consumed test food containing 100 mg PIC for 2 weeks. Additionally, the influence of PIC on mitochondrial biogenesis and fatty acid accumulation was investigated in cell experiments.

## 2. Materials and Methods

### 2.1. Cell Culture and Sample Preparation

C2C12 mouse skeletal muscle cells were cultured as described previously [32]. When cells reached 90–100% confluency, the medium was replaced with a differentiation medium, containing 2% heat inactivated horse serum, and differentiation was initiated. The differentiation medium was replenished every 3 d. After 6 d, the cells underwent differentiation and formed myotubes. Initially, PIC or RES was dissolved in dimethyl sulfoxide (DMSO) and diluted to the desired concentration using the differentiation medium. Passion fruit seed extract (PFSE) was prepared as described previously [47] and dissolved in DMSO. The final concentration of DMSO was kept at 0.1% under all conditions. For fatty acid detection, oleic acid was dissolved in 100 mg/mL bovine serum albumin (BSA) in phosphate-buffered saline (PBS) and diluted to 1 mM in a 1:1 mixture of PBS and differentiation medium. The cells were exposed to 1 mM oleic acid for 1 h. No cytotoxic effects were observed under these conditions.

### 2.2. RNA Isolation, cDNA Synthesis, and Quantitative Real-time PCR

Total RNA was extracted from C2C12 myotubes and cDNA was synthesized as described previously [32]. The amplification of cDNA was performed on a LightCycler 96 Real-Time PCR system (Roche Molecular Diagnostics, Basel, Switzerland) using the KAPA SYBR FAST qPCR Master Mix (Kapa Biosystems, Cape Town, South Africa), following the guidelines provided by the manufacturer. The levels of *Sirt1* mRNA expression were normalized to those of *Gapdh*. The sequences of the gene-specific primers are shown in Table 1.

### 2.3. Western Blotting for Quantification of SIRT1 Protein

Western blotting and antibody detection were performed following the methods reported by Kawakami et al. [47]. Briefly, C2C12 cells were lysed in RIPA buffer, and the concentration of the extracted protein was measured. Proteins (20 μg) were separated using 10% SDS-PAGE and then transferred to PVDF membranes. The membranes were first blocked using 3% BSA (for SIRT1) or 5% skim milk (for GAPDH) in Tris-buffered saline with 0.1% Tween-20 (TBS-T) for 1 h. Subsequently, they were incubated overnight at 4°C with rabbit anti-SIRT1 antibodies (Cat. No. 3931; 1:1000 in 3% BSA/TBS-T; Cell Signaling Technology, Danvers, MA, USA) or rabbit anti-GAPDH antibodies (Cat. No. 2118; 1:5000 in 5% skim milk/TBS-T; Cell Signaling Technology). After washing with TBS-T, the membranes were incubated with horseradish peroxidase-conjugated anti-rabbit IgG (Cat. No. 7074S, Cell Signaling Technology) for SIRT1 (1:1000 in 5% skim milk) or for GAPDH (1:5000 in 5% skim milk), for 1 h at 20–25 °C. Immunoreactive bands were detected using an ECL Prime (GE Healthcare, Chalfont St. Giles, UK). Band intensities were quantified using the ImageJ, with values normalized to that of GAPDH.

### 2.4. Quantification of Mitochondrial DNA

Total DNA was isolated from C2C12 myotubes using the QIAamp DNA Mini Kit (QIAGEN), following the guidelines provided by the manufacturer. Mitochondrial DNA (mtDNA) and nuclear DNA were quantified, as described in Section 2.2. The sequences of primers used for mtDNA amplification are listed in Table 2.

### 2.5. Detection of Fatty Acid Accumulation

C2C12 cells were cultured and differentiated in 96-well black plates, and the cells were treated with 1 mM oleic acid for 1 h at 20–25 °C. The medium was then replaced with a differentiation medium with or without PIC, and the cells were incubated for 48 h. For cellular neutral lipid detection, the cells were washed twice with PBS and stained with 100 ng/mL Nile red in PBS. The cells were then washed twice with PBS and subjected to fluorometric analysis using a microplate fluorescence reader at excitation and emission wavelengths of 486 nm and 528 nm, respectively. For normalization, total protein was extracted from each well, and Nile red fluorescence was normalized to the protein content.

### 2.6. Clinical Trial Design

A clinical trial was conducted in accordance with the ethical principles of the Declaration of Helsinki (revised in 2013) and the Ethical Guidelines for Life Sciences and Medical Research Involving Human Subjects (Ministry of Education, Culture, Sports, Science, and Technology; Ministry of Health, Labor, and Welfare; and Ministry of Economy, Trade, and Industry, Japan). The study was approved by the Institutional Review Board of Chiyoda Paramedical Care Clinic (IRB No. 15000088; approval date: 18 August 2023). Participants were fully informed of the purpose, details, and methods of the study and provided informed consent prior to participating. Participants were recruited and managed by the CPCC Company Ltd., and measurements were conducted at the Chiyoda Paramedical Care Clinic. The study was carried out between September and November 2023. The study protocol was registered in the UMIN-CTR (UMIN ID: UMIN000052082).

### 2.7. Participants

This study selected participants who met the following inclusion criteria but did not meet the exclusion criteria.

Inclusion criteria: (1) males and females > 20 but <70 years old when informed consent was given; (2) individuals with a BMI ≥ 20; and (3) individuals who have received enough explanation, understood the purpose of the study, and can provide informed consent.

Exclusion criteria: (1) individuals who take foods with functional claims or foods or supplements containing polyphenols more than three times a week, or will take them for the test period; (2) individuals who are aware of severe symptoms such as irregular menstruation, menstrual cramps, and symptoms related to menopausal disorders; (3) individuals who participated in other clinical studies in the past 4 weeks, or will participate during the test period; (4) individuals who are heavy alcohol drinkers; (5) individuals with a history of hepatopathy, kidney damage, heart disease, or gastrointestinal disease at present or within the past 5 years; (6) individuals who are allergic to foods or medicines; (7) females who are or are possibly pregnant, or are lactating; (8) individuals who donated a total of more than 200 mL whole blood or blood components in the past 1 month; (9) males who donated a total of more than 400 mL whole blood in the past 3 months; (10) females who donated a total more than 400 mL whole blood in the past 4 months; (11) males who have donated a total of more than 1200 mL blood in the past 12 months, including the test period; (12) females who have donated a total of more than 800 mL blood in the past 8 months, including the test period; and (13) individuals judged inappropriate for this study by the principal investigator.

### 2.8. Test Food

The test food was a drink (200 mL; 33 kcal) containing PIC from passion fruit seed extract. Similarly, the placebo food was a drink (200 mL; 29 kcal) with the same composition as the test food, except that it did not contain passion fruit seed extract. The participants consumed three packs of the test or placebo drink daily for two weeks; the amount of PIC in the test food was 100 mg/d. The nutrient composition of the test and placebo food are shown in Table 3.

### 2.9. Experimental Protocol

This was a randomized, double-blind, placebo-controlled, parallel-group comparison trial. This study was designed to investigate the effects of test foods through subgroup analyses by age, BMI, and sex. Therefore, the target sample size was set at 300, divided into five blocks by age (20 s–60 s) and three blocks by BMI (20.0 to 24.9, 25.0 to 29.9, and ≥30 or over). Ten males and ten females were included in each block.

At the first clinic visit, the participants were informed about the study, their height and weight were measured, and their BMI was calculated. After a medical interview, participants who met the criteria and were judged by the principal investigator to be eligible to participate in the study were selected. Based on sex, age, and BMI, participants were divided into two groups using the stratified randomization method. Subsequently, an independent controller assigned each group to either the test or placebo food. The assigned test food information was kept confidential until analysis was completed. During the study, the participants were instructed to not make any major lifestyle changes, such as excessive exercise or overeating. Any changes in the physical condition of participants or missing a dose of the test or placebo food were recorded. One and two weeks after consuming the test or placebo food, the participants visited the clinic without eating breakfast, and blood samples were collected.

### 2.10. Measurement of SIRT1 mRNA Levels from Blood Samples

For ethical reasons, this study examined SIRT1 expression in whole blood samples. Whole blood from each participant was collected in PAXgene Blood RNA Tubes (BD Biosciences, Franklin Lakes, NJ, USA) and preserved at −20 °C until further analysis. Total RNA was isolated using the PAXgene Blood RNA Kit (QIAGEN), following the guidelines provided by the manufacturer. cDNA synthesis was performed as described in Section 2.2. Subsequent real-time PCR was carried out using FastStart Essential DNA Probe Master Mix (NIPPON Genetics Co., Ltd., Tokyo, Japan) according to the manufacturer’s protocol. The levels of SIRT1 mRNA expression were normalized to that of *GAPDH*. The sequences of the gene-specific primers are listed in Table 4.

### 2.11. Statistical analysis

Data from in vitro assays are expressed as the mean ± standard deviation (SD). For comparing three or more groups, data were analyzed using Tukey’s multiple comparison test or Dunnett’s multiple comparison test. Student’s *t*-test was used to compare the differences between two groups. For the clinical data, participant background data are presented as the mean ± SD, whereas other data are presented as the mean ± standard error of the mean (SE). Differences between groups were analyzed using analysis of covariance (ANCOVA), with the value at baseline (0 week) as a covariate. Statistical analyses were performed using SPSS software version 26 (IBM Corp., Armonk, NY, USA). Statistical significance was set at *p* < 0.05.

## 3. Results

### 3.1. PIC Upregulated the mRNA and Protein Expression of Sirt1

**Figure 1:**
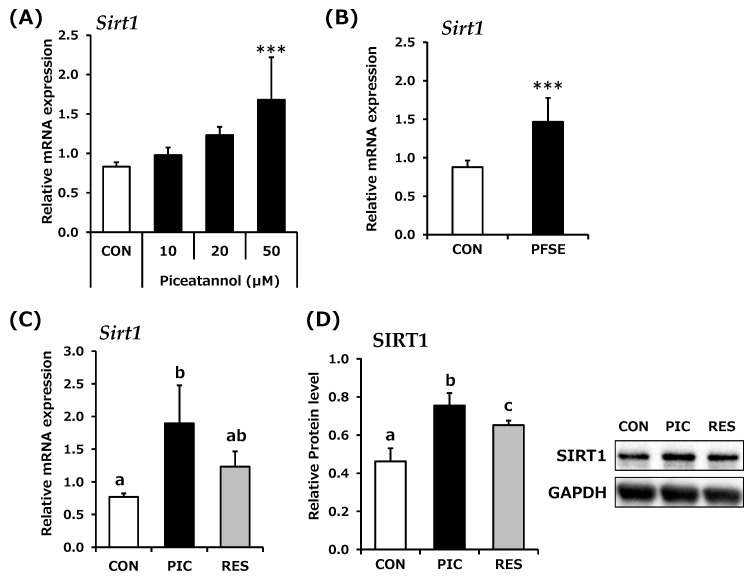
Effect of piceatannol (PIC) on SIRT1 expression in C2C12 myotubes. (**A**) Cells were exposed to 0.1% DMSO (CON; white bar) or PIC at concentrations of 10, 20, and 50 µM (black bar) for 6 h (*n* = 6), and *Sirt1* mRNA expression was measured and normalized to that of *Gapdh*. *** *p* < 0.001 vs. CON (Dunnett’s test). (**B**) Cells were exposed to 0.1% DMSO (CON; white bar) or passion fruit seed extract (PFSE, containing 20 μM PIC; black bar) for 6 h (*n* = 8), and *Sirt1* mRNA expression was measured and normalized to that of *Gapdh*. *** *p* < 0.001 vs. CON (Student’s *t*-test). (**C**) Cells were exposed to 0.1% DMSO (CON; white bar), 50 µM PIC (black bar), or 50 µM RES (gray bar) for 6 h (*n* = 4), and *Sirt1* mRNA expression was measured and normalized to that of *Gapdh*. Significant differences were identified using Tukey’s HSD test. Different letters indicate significance (*p* < 0.05). (**D**) Following a 24 h treatment with 0.1% DMSO (CON; white bar), 50 µM PIC (black bar), or 50 µM RES (gray bar), the relative SIRT1 protein level was normalized to that of GAPDH (*n* = 6). The right panel of (**D**) depicts a typical blot image showing treatments with the control, 50 µM PIC, and 50 µM RES. Tukey’s HSD test was utilized to ascertain significant differences between treatments, with different letters indicating significance (*p* < 0.05).

To examine the effect of PIC on *Sirt1* mRNA expression in skeletal muscle cells, C2C12 myotubes were subjected to quantitative real-time PCR. After 6 h of PIC treatment, a PIC concentration-dependent upregulation of *Sirt1* expression was observed (Figure 1A). The stimulation of PFSE, containing 20 μM PIC, also significantly upregulated SIRT1 mRNA expression, and the effect was similar to that after stimulation with 20 μM PIC. The effect of PIC on *Sirt1* expression was compared with that of RES. After 6 h of stimulation, PIC significantly induced *Sirt1* mRNA expression, whereas RES did not significantly affect *Sirt1* mRNA levels. Moreover, *Sirt1* expression following PIC stimulation was 1.5-fold higher than that following RES stimulation, indicating a significant increasing trend (*p* = 0.07; Figure 1B). The effects of PIC and RES on SIRT1 protein expression were also examined. Both PIC and RES stimulation for 24 h increased SIRT1 protein levels (Figure 1C). Moreover, PIC significantly increased the expression of *Sirt1* by approximately 1.2-fold compared with that induced by RES (*p* = 0.01; Figure 1C).

### 3.2. PIC Enhanced Mitochondrial Biogenesis and Boosted Antioxidant Marker Expression

**Figure 2:**
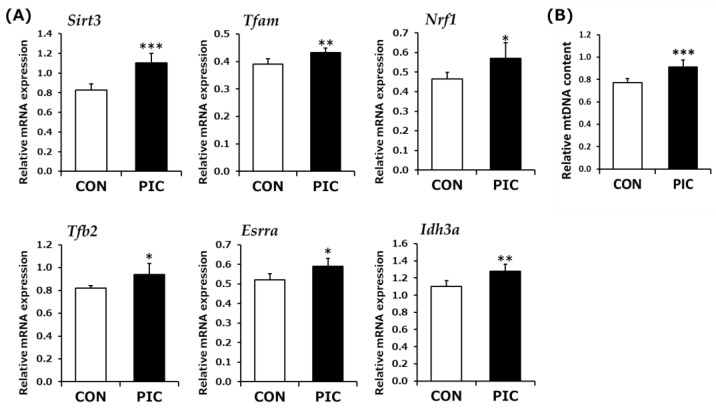
Effect of piceatannol (PIC) on mitochondria-related gene expression and mtDNA copy number in C2C12 myotubes. (**A**) Cells were exposed to 0.1% DMSO (CON; white bar) or 50 µM PIC (black bar) for 24 h (*n* = 5–6). Mitochondrial gene mRNA levels were quantified and normalized to those of *Gapdh*. (**B**) Cells were exposed to 0.1% DMSO (CON; white bar) or 20 µM PIC (black bar) for 48 h (*n* = 7). The mitochondrial-to-nuclear DNA ratio (mtDNA/nDNA) was determined via qPCR after total DNA extraction. * *p* < 0.05, ** *p* < 0.01, *** *p* < 0.001 vs. CON (Student’s *t*-test).

SIRT1 enhances the expression of genes involved in mitochondrial biogenesis [44]. Based on the observed increase in SIRT1 levels in response to PIC, we further investigated whether PIC enhanced the expression of mitochondria-related genes in C2C12 myotubes. After 24 h of treatment, PIC upregulated the expression of various mitochondrial genes, including regulatory genes such as *Sirt3*, *Esrra*, *Nrf1*, *Tfam*, and *Tfb2*, as well as the TCA cycle gene *Idh3a* (Figure 2A). PIC upregulated the expression of genes associated with mitochondria; therefore, mitochondrial content was measured as the mtDNA copy number to verify whether PIC affected mitochondrial mass. As shown in Figure 2B, a significant increase in mtDNA levels was observed after treatment with PIC for 48 h.

**Figure 3:**
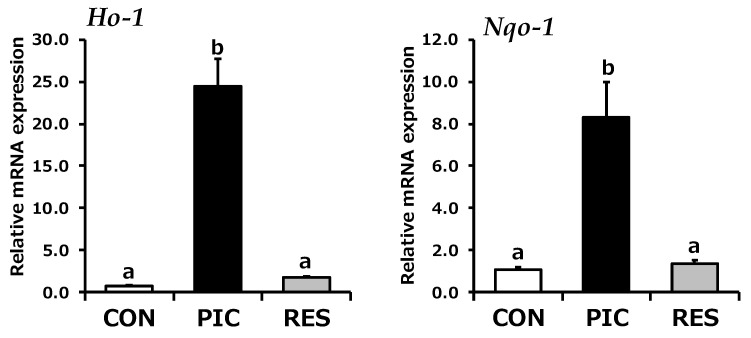
Effect of PIC on *Ho-1* and *Nqo-1* mRNA expression in C2C12 myotubes. Cells were exposed to 0.1% DMSO (CON; white bar), 50 µM PIC (black bar), or 50 µM RES (gray bar) for 6 h (*n* = 4), and *Ho-1* and *Nqo-1* mRNA levels were quantified and normalized to those of *Gapdh*. Significant differences were identified using Tukey’s HSD test, with different letters indicating significance (*p* < 0.05).

Given the key role of the mitochondria in energy production and the associated increase in ROS levels, evaluating the mechanism by which PIC affects cellular antioxidant defense systems is crucial. In the current study, we reconfirmed the effects of PIC treatment on the levels of mRNA expression of the antioxidant enzyme HO-1 and further examined its effect on the antioxidant enzyme NAD(P)H quinone dehydrogenase-1 (NQO-1) to provide a more comprehensive understanding of its effect on cellular antioxidant defense systems. After 6 h of stimulation, PIC significantly induced *Ho-1* and *Nqo-1* mRNA expression, whereas RES did not significantly affect the mRNA expression of these genes. The mRNA expression of *Ho-1* and *Nqo-1* in cells exposed to PIC was approximately 14- and 6-fold higher than that in RES-stimulated cells, respectively (Figure 3).

### 3.3. PIC Upregulated the Expression of Genes Involved in Fatty Acid Utilization and Suppressed Fatty Acid Accumulation

**Figure 4:**
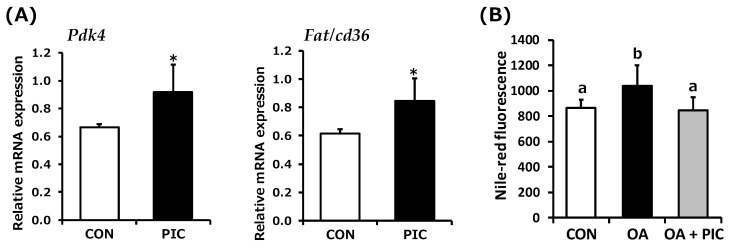
Effect of piceatannol (PIC) on the mRNA level of fatty acid utilization genes and fatty acid accumulation in C2C12 myotubes. (**A**) Cells were exposed to 0.1% DMSO (white bar) or 50 µM PIC (black bar) for 24 h (*n* = 5–6). Gene expression related to fatty acid utilization was quantified and normalized to that of *Gapdh*. * *p* < 0.05. (**B**) Cells were treated with 1 mM oleic acid (OA) or left untreated for 1 h. Subsequently, cells were incubated with or without 50 μM PIC for 48 h. Cells in the control group (CON) were treated with 0.1% DMSO, without OA treatment. Cells in the OA group were treated with 1 mM OA, whereas those in the OA+PIC group were treated with both 1 mM OA and 50 μM PIC. Intracellular lipid droplets were quantified using Nile red staining. Significant differences were identified using Tukey’s HSD test, with different letters indicating significance (*p* < 0.05).

Similar to mitochondria-related genes, SIRT1 also increases the expression of genes involved in fatty acid utilization [48]. Hence, we next investigated whether PIC upregulates these genes. At 24 h posttreatment, an increase in the expression of genes involved in fatty acid utilization, such as *Pdk4* and *Fat/cd36*, was observed (Figure 4A). The effect of PIC treatment on fatty acid accumulation was investigated using oleic acid, and the results revealed that a 48 h stimulation with PIC significantly suppressed oleic acid-induced fat accumulation (Figure 4B).

### 3.4. PIC Upregulated SIRT1 Expression in Human Whole Blood

**Figure 5:**
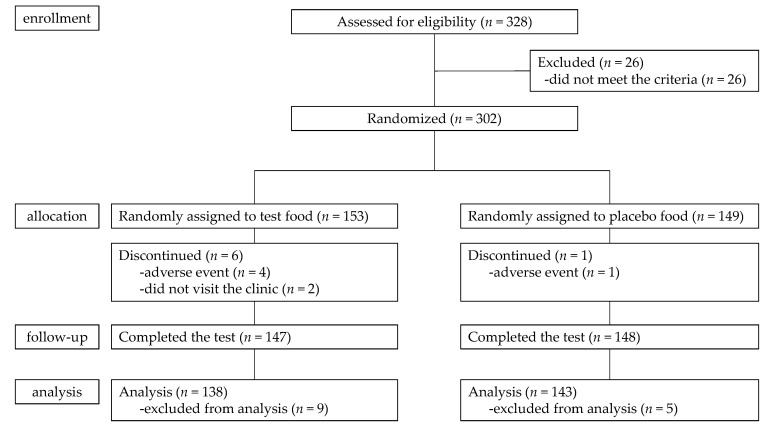
Flow chart of the clinical trial.

A clinical study was conducted to verify whether PIC treatment induced SIRT1 expression in humans. A flow diagram of this study is presented in Figure 5. A total of 302 participants were selected for the study and allocated to the test (PIC group) or placebo (placebo group) food group. Seven participants dropped out during the study period due to the following reasons: did not visit the clinic (PIC group, *n* = 2); had COVID-19 infection (PIC group, *n* = 3); had high blood pressure (placebo group, *n* = 1); or had urticarial symptoms (PIC group, *n* = 1); hence, 295 participants completed the study. After a careful review of their records, 14 participants were excluded from the analysis for the following reasons: low intake of test food (<85% in the test period; PIC group, *n* = 1); failure to maintain fasting on the morning of the first measurement day (placebo group, *n* = 1; PIC group, *n* = 3); or intake of drugs for the treatment of disease, or treatment of adverse events during the study period (placebo group, *n* = 4; PIC group, *n* = 5). Therefore, 281 participants were included in the efficacy evaluation. Their characteristics are shown in Table 5. No significant differences were observed in age, height, body weight, or BMI between the groups.

The results of SIRT1 mRNA expression are shown in Table 6. After one week of intake, SIRT1 expression was significantly higher in the PIC group than in the placebo group.

Subgroup analyses were performed to investigate the differences in SIRT1 expression based on age, BMI, and sex. Subgroup analysis by age showed that SIRT1 expression was significantly higher in the PIC group than in the placebo group for participants at 60–69 years of age (Table S1). BMI subgroup analysis showed that SIRT1 expression was significantly higher in the PIC group in participants with a BMI of 30 or above after 1 week of intake (Table S2). When stratified by sex, SIRT1 expression was significantly higher in the PIC group at 1 week in females only (Table S3). Further analysis according to BMI showed that SIRT1 expression was significantly higher in the PIC group in males with a BMI of 30 or higher and in females with a BMI of 20–25 after 1 week of intake (Table S3). In addition, after stratification based on the presence or absence of menopause, SIRT1 expression was significantly higher in the PIC group in postmenopausal females (Table S4).

During the study, 24 adverse events occurred in 23 participants, whereas no serious adverse events were observed. These adverse events included back pain, arthralgia, gastric distress, stomatitis, acute cystitis, fever, viral gastritis, abdominal pain, diarrhea, headache, COVID-19, hives, itching, chills, acne vulgaris, dizziness, and high blood pressure. Cases of urticarial symptoms (placebo group, *n* = 1; PIC group, *n* = 1) were considered by the investigator to be causally related to the intake of the test food but were transient, and symptoms resolved quickly during the study period. The other 22 adverse events were determined to be unrelated to the intake of the test food.

## 4. Discussion

In this study, PIC as well as PFSE increased *Sirt1* mRNA and protein levels in C2C12 myotubes. Previous studies on the SIRT1-inducing effects of PIC reported various findings. These included a concentration-dependent increase in SIRT1 levels in THP-1 cells following PIC stimulation [47], an increase in hepatic *Sirt1* expression in mice fed a high-fat diet after receiving PIC for 4 weeks [49], and the restoration of diminished *Sirt1* expression in a cerebral ischemia–reperfusion injury mouse model after the oral intake of PIC [50]. Taken together, these findings suggest that PIC is a potent compound that can improve health by increasing SIRT1 expression in various tissues.

This study demonstrated that PIC enhances the expression of genes related to mitochondrial biogenesis, such as *Sirt3*, *Tfam*, *Nrf1*, *Tfb2*, and *Esrra*, increasing mtDNA content in C2C12 cells. The activation of SIRT1 leads to the deacetylation of PGC-1α [13], which interacts with various transcription factors to induce mitochondrial biogenesis [48,51,52]. In the present study, we also found that PIC enhanced the expression of genes involved in fatty acid utilization, such as *Pdk4* and *Fat/cd36*, which leads to a reduction in the accumulation of neutral fats induced by oleic acid. The deacetylation of PGC-1α enhances the expression of fatty acid utilization genes, including *Pdk4* and *Fat/cd36* [48]. SIRT1 also regulates the nuclear receptor peroxisome proliferator-activated receptor-α (PPARα), thereby modulating lipid homeostasis [53]. Our previous studies reported that PIC intake in humans enhances energy expenditure from fat, both at rest and during moderate physical activity [38,39]. Considering these studies and our present findings, we propose that the consumption of PIC not only activates SIRT1, which in turn regulates PGC-1α and PPARα, but also simultaneously promotes mitochondrial biogenesis and enhances fatty acid utilization at the cellular level. Together, these processes may contribute to an increase in mitochondrial density and fat consumption, collectively leading to an increase in fat consumption throughout the body.

The effect of PIC on SIRT1 induction was compared with that of RES, and PIC enhanced SIRT1 level to an extent equivalent to or greater than that achieved with RES. In addition, our current study reconfirmed that PIC significantly increased not only *Ho-1* expression [32], a process that is NRF2-dependent [54], but also *Nqo-1* expression compared with RES. A recent study reported that NQO-1 physically interacts with SIRT1 and modulates its activity [55]. NQO-1 produces NAD^+^, which is essential for SIRT1 activity. These findings suggested that an upregulation in *NQO-1* expression can enhance the activity of SIRT1. Our previous study found that intact PIC has a higher bioavailability than RES [56] and that both isorhapontigenin and methylated metabolites of PIC upregulate SIRT1 expression [47]. Thus, PIC may be a more potent polyphenol than RES for activating SIRT1. Some polyphenols, such as cocoa polyphenol extract or quercetin, were reported to induce SIRT1 expression [57,58]. Further studies on the differences in SIRT1 induction effects between PIC and other polyphenols will clarify the characteristics of PIC effects.

SIRT1 is effective against various metabolic diseases. For instance, mice overexpressing *Sirt1* and fed a high-fat diet exhibited less lipid-induced inflammation and improved glucose tolerance compared with those in their nontransgenic counterparts [59]. Moreover, clinical trials with obese participants demonstrated that calorie restriction increased SIRT1 expression in peripheral blood mononuclear cells and significantly reduced body weight, BMI, as well as the levels of free fatty acids, fasting insulin, and inflammatory markers [60]. Although further human clinical trials are needed to verify our findings, PIC, as a SIRT1 activator, can potentially promote mitochondrial biogenesis and enhance fatty acid consumption through SIRT1 induction. Further investigation of the involvement of SIRT1 in the promotion of mitochondrial biosynthesis and fat metabolism by PIC will further clarify the mechanism of action of PIC.

This study showed that compared with the placebo, PIC consumption significantly increased SIRT1 expression in participants with no apparent disease, other than a high BMI. No significant increase in SIRT1 level was observed after 2 weeks of PIC intake, and although the reason for this has not been clarified, further investigation of the PIC dosage and its treatment duration will improve our understanding of the SIRT1 induction effect of PIC. We previously found that the intake of PIC from passion fruit seed for 1 or 2 weeks increases fat burning in healthy individuals [38,39]. Considering that SIRT1 promotes fat burning, the duration of SIRT1 induction observed in this study is considered reasonable. A clinical trial reported that the ingestion of 500 mg RES for 4 weeks resulted in an increase in blood SIRT1 expression as a metabolic regulatory marker in patients with type 2 diabetes mellitus and coronary heart disease [45]. In this study, PIC ingestion at a dose lower than that of RES resulted in the induction of SIRT1 expression. In animal studies, the blood concentration of the unchanged form of PIC was reported to be higher than that of RES when co-administrated [56]. In addition, metabolites of PIC have also been shown to have a SIRT-inducing effect [47], suggesting that PIC is a more effective ingredient than RES in inducing SIRT1 expression. PIC can be detected in blood after a single intake of food containing 100 mg PIC [61], suggesting that SIRT1 expression is induced by the action of PIC and its metabolites on cells in blood such as peripheral blood mononuclear cells.

In addition, the subgroup analysis of blood SIRT1 expression by age and BMI showed a significant effect of PIC in the older age and higher BMI subgroups. Obese subjects have been reported to have lower *Sirt1* mRNA expression in the subcutaneous adipose tissue than normal-weight and overweight participants [62], and *Sirt1* expression in the brain, liver, skeletal muscle, and WAT was shown to decrease in aging mice [63]. Our findings suggested that PIC may rescue the decline in SIRT1 expression in subjects with high BMI or advanced age. Moreover, the results of the clinical trial showed differences in the effects of PIC on SIRT1 induction between males and females. In particular, differences were observed in the effects of PIC depending on menopausal status, suggesting a possible influence of sex hormones on the biological effects of PIC. Of note, *Sirt1* expression was found to be decreased in the aorta of ovariectomized mice [64], while estradiol, a female sex hormone, was reported to affect SIRT1 expression [65]. Moreover, PIC acts as a phytoestrogen, phosphorylating hormone-sensitive lipase by activating the G protein-coupled estrogen receptor and reducing fat accumulation [66]. These findings suggested that the phytoestrogenic action of PIC may affect SIRT1 expression in postmenopausal females.

This clinical study had some limitations. First, as SIRT1 expression has been reported to vary depending on the meal consumed before measurement [67], participants were required to fast on the morning of the measurement day. However, participants had no dietary restriction on the day before the measurement day. Hence, a more severe dietary control may clearly demonstrate the effect of the intervention. Second, the dosage and duration of intervention need to be further examined. 

In this study, we focused on SIRT1, but other sirtuins also affect the aging process and metabolic function, such as SIRT6 [68] and SIRT7 [69]. Little is known about the effects of PIC on such other sirtuins, and further investigation will improve our understanding of the action of PIC.

## 5. Conclusions

We demonstrated that PIC increases SIRT1 levels and upregulates the expression of mitochondrial biogenesis and fatty acid utilization genes in skeletal muscle cells. This leads to an increased amount of mitochondrial DNA and reduced fatty acid accumulation. In the clinical trial, SIRT1 expression in the whole blood was significantly higher in the PIC than in the placebo group. These findings indicate that PIC enhances SIRT1 expression, leading to increased mitochondrial activity and fat consumption, and may contribute to preventing various age-related diseases.

## Figures and Tables

**Table 1:** Sequences of the PCR primers used for real-time PCR in C2C12 cells.

Gene	Forward Sequence (5′-3′)	Reverse Sequence (5′-3′)
*Gapdh*	TCCAGTATGACTCCACTC	ATTTCTCGTGGTTCACAC
*Sirt1*	TCGTGGAGACATTTTTAATCAGG	GCTTCATGATGGCAAGTGG
*Ho-1*	AGGCTAAGACCGCCTTCCT	TGTGTTCCTCTGTCAGCATCA
*Nqo-1*	AGAGAGTGCTCGTAGCAGGAT	GTGGTGATAGAAAGCAAGGTCTT
*Pdk4*	CACATGCTCTTCGAACTCTTCAAG	TGATTGTAAGGTCTTCTTTTCCCAAG
*Fat/cd36*	GATGACGTGGCAAAGAACAG	TCCTCGGGGTCCTGAGTTAT
*Sirt3*	GCCTGCAAGGTTCCTACTCC	TCGAGGACTCAGAACGAACG
*Idh3α*	AGGACTGATTGGAGGTCTTGG	ATCACAGCACTAAGCAGGAGG
*Tfam*	CACCCAGATGCAAAACTTTCAG	CTGCTCTTTATACTTGCTCACAG
*Nrf1*	CCACGTTGGATGAGTACACG	CTGAGCCTGGGTCATTTTGT
*Tfb2*	TTTTGGCAAGTGGCCTGTGA	CCCCGTGCTTTTGACTTTTCTA
*Esrrα*	AAGACAGCAGCCCCACTGAA	ACACCCAGCCCAGCACCT

**Table 2:** Sequences of the PCR primers used for mitochondrial DNA quantification in C2C12 cells.

Gene	Forward Sequence (5′-3′)	Reverse Sequence (5′-3′)
*Genome DNA (LPL)*	GGATGGACGGTAAGAGTGATTC	ATCCAAGGGTAGCAGACAGGT
*Mitochondrial Gene (NDH-I)*	CCCATTCGCGTTATTCTT	AAGTTGATCGTAACGGAAGC

**Table 3:** Nutrient composition of test food and placebo food (per daily intake).

	Test Food	Placebo Food
Protein (g)	0	0
Fat (g)	0	0
Carbohydrate (g)	39	36
NaCl (g)	1.2	1.1
Piceatannol (mg)	100	0

**Table 4:** Sequences of the PCR primers and probes used for real-time PCR in the clinical trial.

Gene	Forward Sequence (5′-3′)	Reverse Sequence (5′-3′)	Probe (5′-3′)
*GAPDH*	CCATCTTCCAGGAGCGAGAT	GGGCAGAGATGATGACCCTT	AGTCCACTGGCGTCTTCACCACCAT
SIRT1	ACTGGAGCTGGGGTGTCTG	CATCGCTTGAGGATCTGGAAGA	CTACAGCAAGGCGAGCATAAATACCATCCC

**Table 5:** Baseline characteristics of the study participants.

	PIC Group (*n* = 138)	Placebo Group (*n* = 143)	*p*-Value
Age (years)	44.9 ± 13.8	44.8 ± 13.1	0.981
Sex	69 male/69 female	72 male/71 female	
Height (cm)	164.6 ± 8.6	163.8 ± 8.6	0.441
Body weight (kg)	74.9 ± 16.6	72.9 ± 15.0	0.295
Body mass index (kg/m^2^)	27.5 ± 5.1	27.1 ± 4.7	0.436

**Table 6:** SIRT1 mRNA expression in the whole blood of participants in this study.

		0 Week		1 Week		*p*-Value	2 Week		*p*-Value
		AVE	SE	AVE	SE		AVE	SE	
Placebo	*n* = 143	0.989	0.019	0.977	0.019	0.027	0.950	0.018	0.299
PIC	*n* = 138	0.962	0.018	0.997	0.020		0.951	0.018	

## Data Availability

Data supporting the findings of this study are available from the corresponding author upon request.

## Supplementary Materials

The following supporting information can be downloaded at: https://www.mdpi.com/article/10.3390/life14050589/s1, Table S1: Subgroup analysis of SIRT1 expression by age; Table S2: Subgroup analysis of SIRT1 expression according to body mass index (BMI); Table S3: Subgroup analysis of SIRT1 expression by sex and body mass index (BMI); Table S4: Subgroup analysis of SIRT1 expression by menopausal status in female participants.
