# Supplementary material for: Piceatannol Upregulates SIRT1 Expression in Skeletal Muscle Cells and in Human Whole Blood: In Vitro Assay and a Randomized, Double-Blind, Placebo-Controlled, Parallel-Group Comparison Trial
Source: Life (Basel). 2024 May 5;14(5):589. doi: 10.3390/life14050589 (PMC11122325; doi:10.3390/life14050589)
Supplement: Supplementary file 1 [file life-14-00589-s001.zip › Supplementary Table.pdf]

Supplementary Table

Table S1. Subgroup analysis of *SIRT1* expression by age.

		age	0 week		1 week		*p*-value	2 week		*p*-value
			AVE	SE	AVE	SE		AVE	SE	
Placebo	$n = 28$	20-29y	0.943	0.046	0.971	0.043	0.960	0.922	0.041	0.460
PIC	$n = 29$		0.966	0.044	0.990	0.052		0.902	0.044	
Placebo	$n = 27$	30-39y	1.036	0.053	0.961	0.043	0.074	0.947	0.046	0.730
PIC	$n = 28$		0.933	0.035	0.944	0.039		0.905	0.040	
Placebo	$n = 31$	40-49y	0.972	0.037	0.996	0.043	0.690	0.971	0.037	0.500
PIC	$n = 24$		0.971	0.046	1.017	0.051		0.947	0.034	
Placebo	$n = 33$	50-59y	1.057	0.039	1.065	0.036	0.505	1.033	0.041	0.588
PIC	$n = 29$		0.954	0.036	1.027	0.042		0.982	0.037	
Placebo	$n = 24$	60-69y	0.920	0.031	0.854	0.036	0.008	0.846	0.035	0.004
PIC	$n = 28$		0.985	0.037	1.007	0.037		1.020	0.044	

Table S2. Subgroup analysis of *SIRT1* expression according to body mass index (BMI).

		BMI	0 week		1 week		*p*-value	2 week		*p*-value
			AVE	SE	AVE	SE		AVE	SE	
Placebo	*n* = 52	20≦BMI<25	1.000	0.034	1.014	0.036	0.290	0.975	0.033	0.241
PIC	*n* = 47		0.970	0.029	1.025	0.033		0.996	0.034	
Placebo	*n* = 47	25≦BMI<30	0.997	0.030	1.000	0.030	0.356	0.977	0.030	0.429
PIC	*n* = 48		1.000	0.029	1.029	0.030		0.951	0.029	
Placebo	*n* = 44	30≦BMI	0.968	0.034	0.909	0.028	0.039	0.892	0.030	0.055
PIC	*n* = 43		0.910	0.033	0.931	0.038		0.902	0.030	

Table S3. Subgroup analysis of *SIRT1* expression by sex and body mass index (BMI).

		sex and BMI	0 week		1 week		*p*-value	2 week		*p*-value
			AVE	SE	AVE	SE		AVE	SE	
Placebo	*n* = 72	Men	0.993	0.026	0.994	0.026	0.358	0.961	0.024	0.585
PIC	*n* = 69		0.971	0.024	1.008	0.027		0.962	0.024	
Placebo	*n* = 71	Women	0.985	0.028	0.959	0.027	0.018	0.939	0.028	0.351
PIC	*n* = 69		0.953	0.025	0.986	0.029		0.940	0.028	
Placebo	*n* = 25	Men,	0.947	0.043	1.014	0.059	0.949	0.951	0.042	0.603
PIC	*n* = 23	20≦BMI<25	0.965	0.046	1.025	0.047		0.988	0.045	
Placebo	*n* = 25	Men,	1.013	0.044	1.016	0.038	0.876	0.995	0.043	0.602
PIC	*n* = 24	25≦BMI<30	1.012	0.038	1.010	0.042		0.969	0.041	
Placebo	*n* = 22	Men,	1.022	0.046	0.947	0.034	0.028	0.933	0.040	0.151
PIC	*n* = 22	30≦BMI	0.933	0.042	0.987	0.052		0.929	0.038	
Placebo	*n* = 27	Women,	1.049	0.051	1.013	0.045	0.045	0.998	0.051	0.193
PIC	*n* = 24	20≦BMI<25	0.975	0.038	1.024	0.046		1.004	0.052	
Placebo	*n* = 22	Women,	0.978	0.041	0.981	0.048	0.162	0.955	0.043	0.583
PIC	*n* = 24	25≦BMI<30	0.988	0.044	1.048	0.044		0.933	0.043	
Placebo	*n* = 22	Women,	0.914	0.049	0.870	0.043	0.523	0.850	0.046	0.261
PIC	*n* = 21	30≦BMI	0.886	0.050	0.876	0.054		0.874	0.048	

Table S4. Subgroup analysis of *SIRT1* expression by menopausal status in female.

			0 week		1 week		*p*-value	2 week		*p*-value
			AVE	SE	AVE	SE		AVE	SE	
Placebo	*n* = 46	premenopausal	0.979	0.038	0.970	0.036	0.338	0.952	0.038	0.366
PIC	*n* = 45		0.904	0.030	0.933	0.035		0.870	0.029	
Placebo	*n* = 25	postmenopausal	0.997	0.038	0.939	0.039	0.008	0.914	0.040	0.006
PIC	*n* = 24		1.043	0.042	1.079	0.045		1.071	0.050	
